# Identifying obesogenic environment through spatial clustering of body mass index among adults

**DOI:** 10.1186/s12942-024-00376-5

**Published:** 2024-06-26

**Authors:** Kimberly Yuin Y’ng Wong, Foong Ming Moy, Aziz Shafie, Sanjay Rampal

**Affiliations:** 1 Centre of Epidemiology and Evidence Based Practice, Department of Social and Preventive Medicine, Faculty of Medicine, University Malaya, Kuala Lumpur, Malaysia; 2 Department of Geography, Faculty of Social Sciences, University Malaya, Kuala Lumpur, Malaysia

**Keywords:** BMI, Obesity, Obesogenic, Spatial analysis, Spatial clustering

## Abstract

**Background:**

The escalating trend of obesity in Malaysia is surmounting, and the lack of evidence on the environmental influence on obesity is untenable. Obesogenic environmental factors often emerge as a result of shared environmental, demographic, or cultural effects among neighbouring regions that impact lifestyle. Employing spatial clustering can effectively elucidate the geographical distribution of obesity and pinpoint regions with potential obesogenic environments, thereby informing public health interventions and further exploration on the local environments. This study aimed to determine the spatial clustering of body mass index (BMI) among adults in Malaysia.

**Method:**

This study utilized information of respondents aged 18 to 59 years old from the National Health and Morbidity Survey (NHMS) 2014 and 2015 at Peninsular Malaysia and East Malaysia**.** Fast food restaurant proximity, district population density, and district median household income were determined from other sources. The analysis was conducted for total respondents and stratified by sex. Multilevel regression was used to produce the BMI estimates on a set of variables, adjusted for data clustering at enumeration blocks. Global Moran’s I and Local Indicator of Spatial Association statistics were applied to assess the general clustering and location of spatial clusters of BMI, respectively using point locations of respondents and spatial weights of 8 km Euclidean radius or 5 nearest neighbours.

**Results:**

Spatial clustering of BMI independent of individual sociodemographic was significant (p < 0.001) in Peninsular and East Malaysia with Global Moran’s index of 0.12 and 0.15, respectively. High-BMI clusters (hotspots) were in suburban districts, whilst the urban districts were low-BMI clusters (cold spots). Spatial clustering was greater among males with hotspots located closer to urban areas, whereas hotspots for females were in less urbanized areas.

**Conclusion:**

Obesogenic environment was identified in suburban districts, where spatial clusters differ between males and females in certain districts. Future studies and interventions on creating a healthier environment should be geographically targeted and consider gender differences.

## Introduction

Obesity, typically assessed through body mass index as a criterion for body weight in relation to height, is a pressing concern for public health [1]. Approximately 4 million deaths and 120 million disability-adjusted life-years are associated with obesity and it is the second most preventable risk factor globally [2, 3]. Obesity caused financial burdens through increased healthcare expenditures and decreased work productivity, in addition to affecting physical, mental, and social well-being [4, 5]. Obesity results from excess energy intake which is produced when an abundance of food, low physical activity and several environmental factors interact with genetic susceptibility [6]. Genetic factors predisposing individuals to obesity can be attenuated by healthy lifestyle choices [7]. However, healthy lifestyle and food behaviour is challenged by an obesogenic environment [8–10]. The obesogenic environment can influence food and beverage choices, enabling and reinforcing preferences for unhealthy foods, and furthering the unhealthy food environments [11]. The environment plays a pivotal role in managing and preventing obesity, thus, creating a healthy environment should be at the forefront of the public health agenda.

Globally, the increase in the prevalence of obesity has been more pronounced in developing countries, whilst plateauing in developed countries [12]. Nationwide surveys in Malaysia have reported a consistent increase in the prevalence of obesity from 14% in the year 2006 to 17.7% in the year 2015, and 19.7% in the year 2019, with greater increases occurring in areas with developing economies and among the indigenous population [13–15]. Studies among low-income city-dwellers in Kuala Lumpur capital city equivocally reported a higher incidence of obesity than the national average [16, 17]. The disproportionate burden of obesity among different population groups and areas is largely influenced by socioeconomic disparities and exposure to obesogenic environments [18]. Urbanization has led to a lifestyle of eating out due to the greater density of food outlets and time constraints from longer working hours [19, 20]. Overall, 40% of Malaysians consume meals outside of home daily, spending 21% of their household expenditure [21]. Habitual consumption of fast food, processed food, and sugar-sweetened beverages has increased, with an average of 20% of adults consuming fast food at least once a week, while 95% of Malaysian adults reported inadequate fruit and vegetable intake [16, 22–24].

Malaysia is a multiethnic country with geographical distribution of ethnicity across the country. Traditionally, the Malay ethnic and indigenous group resided in rural areas, while the Chinese community concentrated in urban areas due to migration during the colonial period for trade, commerce, and mining. The Indian community resided in estates owned by British colonial authorities or private companies, which have since transformed into suburban areas. As higher prevalence of obesity was observed among non-Chinese ethnic groups [25–28], this could potentially reflect in a higher prevalence of obesity at non-urban areas. However, national surveys in Malaysia found no evidence of differences in the prevalence of obesity between the general urban rural divide [29]. Instead, research among residents of low-income housing at capital city Kuala Lumpur consistently demonstrated an elevated incidence of obesity, particularly those with higher financial resources [16, 17]. Additionally, studies of rural communities from various locations found prevalence of obesity, ranging from 8% to 60.4% [30–33]. These findings emphasized the significance of the local environment in the development of obesity, but evidence of obesogenic environment in Malaysia was limited. Recreational area density, population density, and property value, exhibited protective effects against hospital admission in Kuala Lumpur city [34]. The location of convenience stores and public parks was positively associated with higher body weight [35]. In urban Johor Bahru district, perceived availability of food establishments including supermarkets, grocery stores, convenience stores, fast-food and non-fast-food restaurants, and the affordability of healthy and unhealthy food items were associated with BMI [36]. The density of western fast-food franchises such as KFC, Pizza Hut and McDonalds’ outlets per population in Malaysia was greater than other Asian countries [37]. Therefore, it is imperative to bridge the knowledge gap on the presence of obesogenic environments.

Obesogenic environmental features appeared in practical situations due to common environmental, demographic, or cultural effects shared by neighbouring regions that affect lifestyle [38]. Identifying spatial clusters of obesity could assist governments in locating high-risk areas for targeted public health interventions [39]. Studies from western countries mostly reported obesity clusters at rural, and lower socioeconomic locations whilst, obesity ‘cold spots’ were located in urban areas with higher fruits and vegetable consumption [40–46]. Gender heterogeneity in spatial clustering was revealed in some studies [47–49]. In contrast, evidence on spatial clusters of obesity from developing countries are still limited, though China and India have reported higher obesity prevalence in urban regions [50, 51]. Spatial clustering analysis were commonly conducted as secondary analyses of large national datasets, aggregating weighted values to represent administrative geographical areas [52–54]. However, resource constraints often exist to acquire sufficient samples for estimating prevalence at specific locations in developing countries [53, 55, 56]. Thus, this study aimed to identify obesogenic environments in Malaysia, a middle-income developing country, through the spatial clustering of body mass index among adults from national health surveys, by utilizing point locations and personalized spatial weights.

## Methods

### Study area

**Fig. 1:**
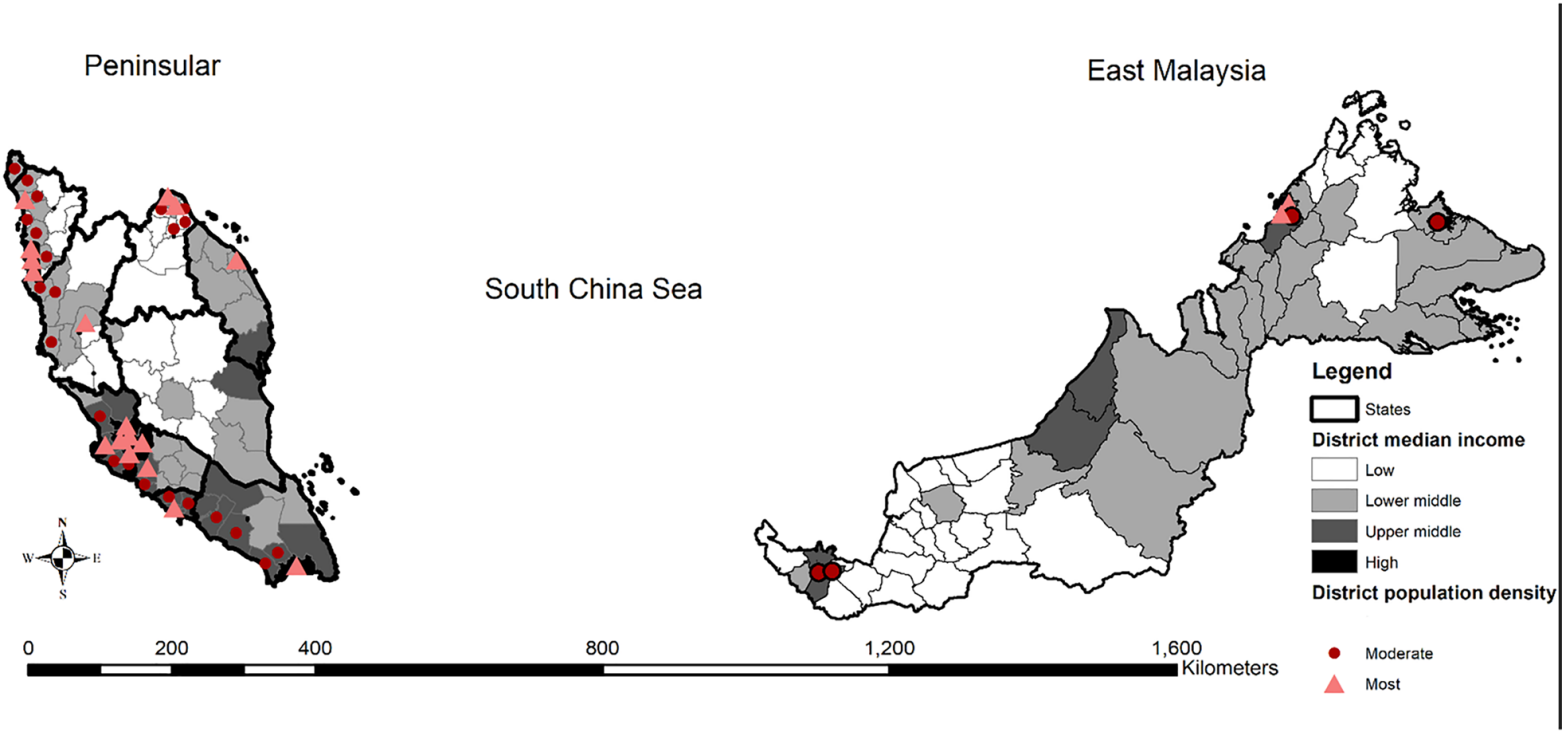
Location of study area in Malaysia

Malaysia is situated in Southeast Asia at coordinates 4.1936° N, 103.7249° E. Geographically, Malaysia is separated into Peninsular Malaysia and East Malaysia, with total areas of 132,265 km^2^ and 198,081 km^2^, respectively. Malaysia comprises of 13 states and three federal territories, with 144 administrative districts. In this present study, district median income represented the socio-economic level of each district, and the district population density represents the degree of urbanization of a district, where high population density was an urban district and moderate population density was suburban district. The population of Malaysia, which was 31.2 million in 2015, with 70% of the population reside in urban areas [57]. Kuala Lumpur is situated on the central west coast of the Peninsular and serves as the capital city. An estimated 20% of total population resides in the surrounding areas of Kuala Lumpur (Fig. 1).

### Study population

This study was a secondary analysis of two nationwide health surveys: the National Health and Morbidity Survey (NHMS) 2015 and the Malaysian Adult Nutrition Survey (MANS) 2014, conducted by the National Institute of Health, Malaysia. Information from both surveys was obtained officially, and details of the methodology were extracted from official technical reports [58, 59]. In brief, both surveys were conducted as a multistage cluster sampling. The allocation of samples was done in proportionate to the size of state population, where states with higher population have higher number of selected enumeration blocks. Twelve living quarters were selected from each selected enumeration block. Face-to-face interviews were conducted to obtain information on socio-demographics, while anthropometry was measured using calibrated machines by trained facilitators. GPS coordinates were entered after each interview using a location detector on a mobile data collection device. In rural areas with no Internet connection or street networks, location coordinates were detected using a Garmin GPS handheld device. Respondents who were more than 60 years old, had no weight or height information, or were from island districts were excluded from this study.

### Sociodemographic

The information of respondents from both surveys was consolidated. Sex (male, female), ethnicity (Malay, Chinese, Indian, Indigenous group, and others), education (primary or unknown, secondary, tertiary), marital status (single, married), occupation (government, private, self-employed, unemployed and others), zone (Peninsular Malaysia, East Malaysia) and urbanity (urban, rural) were categorized as in the primary study. Respondent’s income from both surveys differed, where NHMS 2015 was household income and MANS 2014 was individual income. Hence, the income level variable was separately categorized into quintiles to create an ordinal distribution before they were consolidated into one single variable. Missing information was dummy coded as unknown or other.

### Verification of geographical coordinates

For data privacy, the home locations of the respondents in latitude and longitude coordinates were truncated to three decimal points, equivalent to 100 m on land. The latitude and longitude were subsequently transformed into the Kertau RSO Malaya (Meter) projection system from its original WCS1984 format. The base map was obtained from the Department of Statistics Malaysia based on the census delineation of 2010. The accuracy of the coordinates was manually crosschecked against the district code from the identifier variable. In cases of discrepancies, the coordinates were manually corrected using values from respondents in the same enumeration block.

### Environmental characteristics

The location urbanity of each respondent was obtained from the surveys. Urban area is defined as a gazetted area with a combined population of 10,000 or more with at least 60% of the adult population engaged in non-agricultural activities [60]. The district median household income was sourced from the Malaysian Household Income Survey 2014 [57] to represent the district’s socioeconomic level. The district median household income was categorized into four categories using the Jenks classification from the ArcGIS 10.7 software, to identify low, lower-middle, upper-middle, and high-income districts. The total area of each district was determined, and the population density was calculated as the total district population / area in km^2^. District population density (persons per km^2^) was categorized into three levels: below 150, 150–500 and above 500, to represent the degree of urbanization in a district [60, 61]. The categorization was based on the criteria of local authorities in Malaysia. The criteria stipulated for a District Council is having a total population not exceeding 150,000 people and annual revenue less than RM20 million. Municipal Council refers to local authority in urban or town centre which has a total population exceeding 150,000 people and an annual revenue exceeding RM20 million. City Council/City Hall is a local authority which has been upgraded from municipal council status after having successfully achieved certain criteria which include the total population exceeding 500,000 people and the annual revenue exceeding RM100 million [60].

**Fig. 2:**
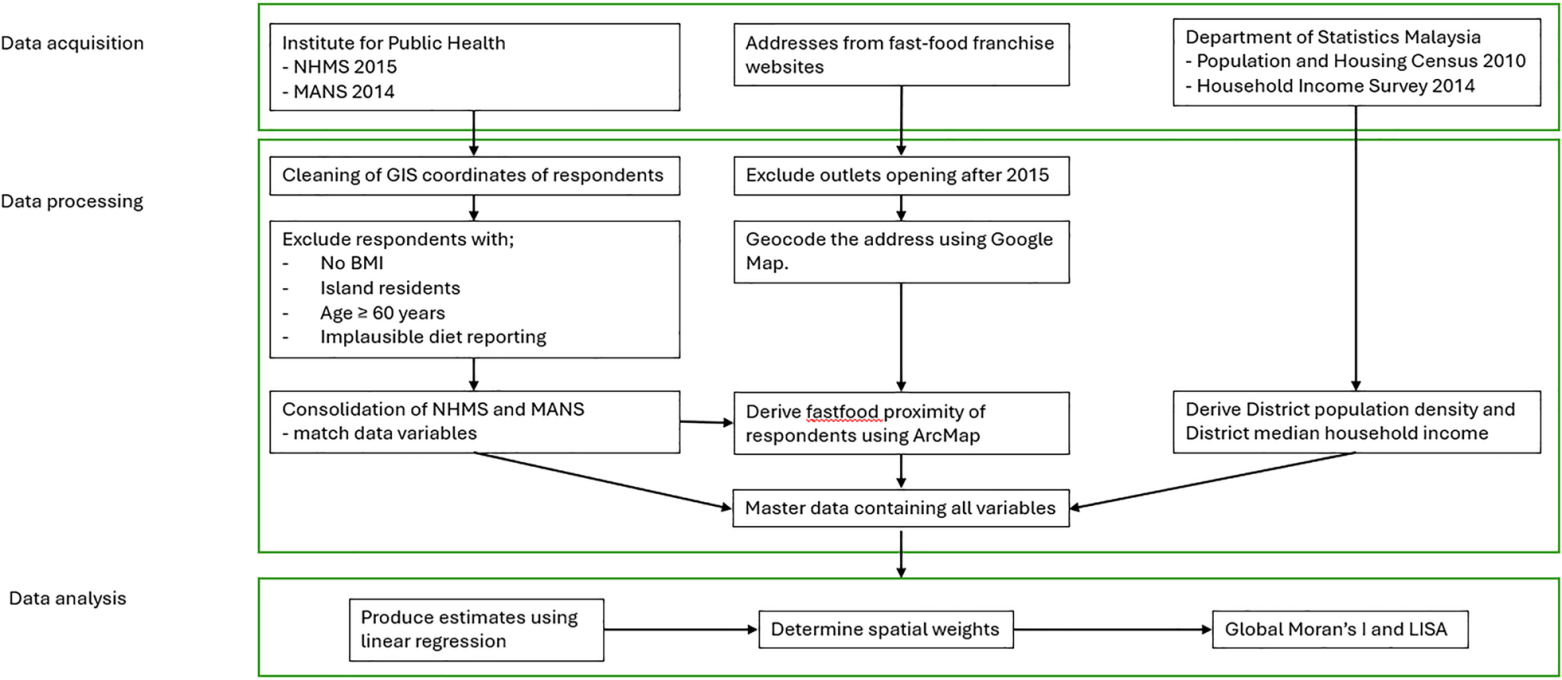
Methodological flowchart for the present study

The addresses of fast-food restaurants were sourced from the official websites of major franchise brands (KFC, McDonalds, Pizza Hut, Dominos, A&W, Marrybrown, and Sugarbun) in January 2018. To reduce the time-lapse between the survey and sampling of fast-food restaurants, each of the fast-food outlets was manually searched on the internet for its opening date. Any fast-food outlets that opened after 2015 were excluded from the analysis. The addresses were then manually geocoded using Google Maps and transformed into the Kertau RSO Malaya Meter coordinate system to facilitate spatial distance calculations. The Euclidean distance between each respondent and the nearest fast-food restaurant was calculated using the proximity function in ArcGIS 10.7. The methodological flowchart of the present study was illustrated in Fig. 2.

### Conceptualization of spatial relationships and areas of influence

The 8 km was selected as the buffer distance to define the area of influence for each respondent. Previous research had reported that 8 km was the furthest distance a person would travel to procure food [62]. Moreover, individuals who mostly travel by car are not limited by their walking distance to the store [63]. The area of influence of each respondent in this present study was dependent on their residence location, instead of pre-fixed areal boundaries using zip codes or government administrative boundaries. The uncertain geographic context problem (UGCoP) occurred when using fixed contextual units, when studying obesity and ignoring the environmental influences experienced by individuals in areas beyond their residential neighbourhood [64]. An individual is not restricted to their residence district to obtain food. Hence, the 8 km distance was assumed to be the most suitable area of influence for this study’s population, which comprised of both urban and rural residents.

**Fig. 3:**
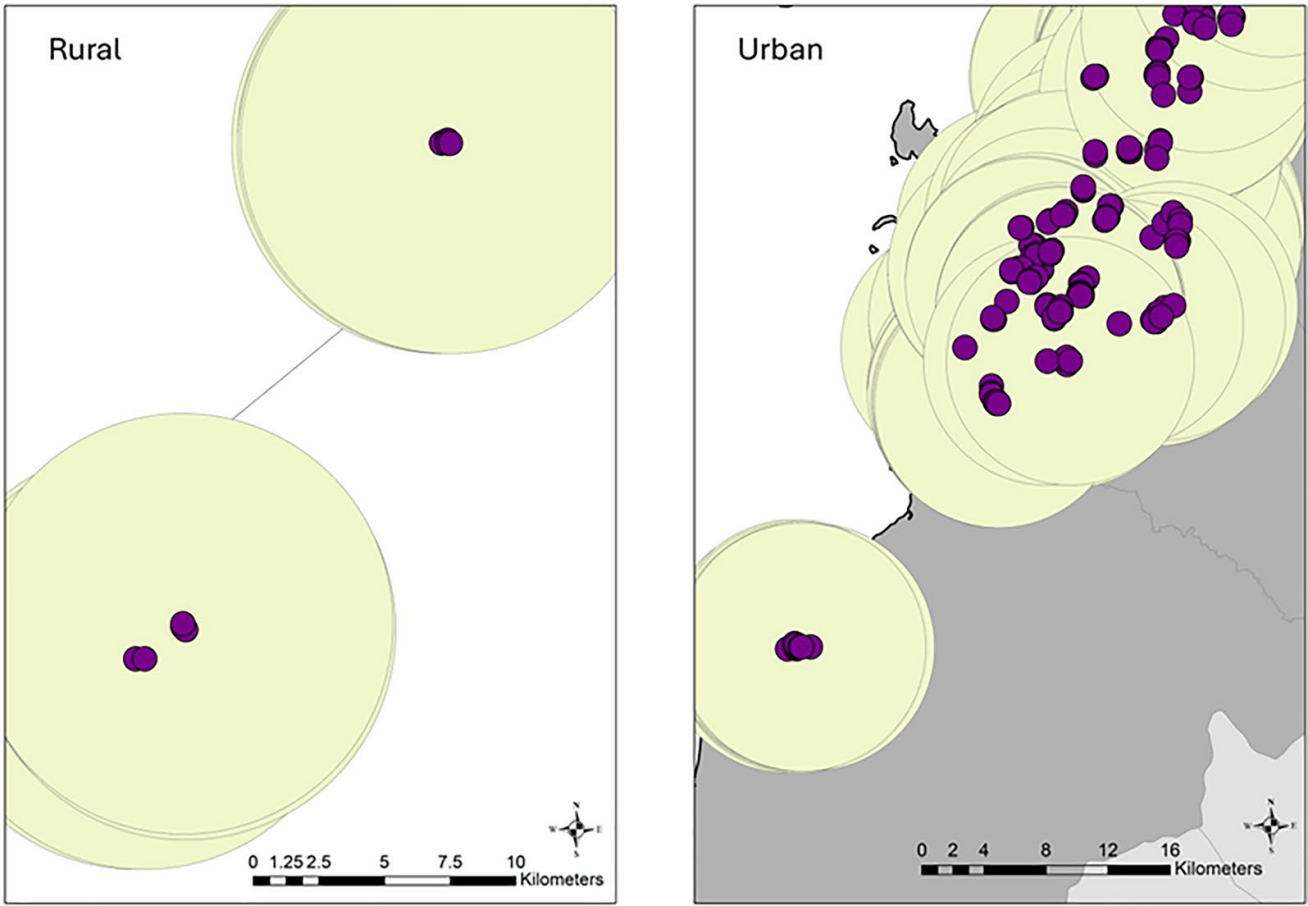
Location of respondents and buffer of 8 km at rural and urban area

The locations of the respondents in MANS and NHMS were irregularly situated where abundant points were found in urban areas, whereas sparse and minimal points were found in rural areas. Within the 8 km radius, 44 (0.3%) respondents in Peninsular had five or fewer neighbours, and 16.2% had fewer than 30 neighbours. In East Malaysia, this number was greater, where 1.0% had five or fewer neighbours and 40.3% had fewer than 30 neighbours within 8 km. The scattered points were included by generating a spatial weight matrix, where points with five or fewer neighbours within an 8 km radius and additional neighbours were included based on the nearest proximity until a minimum of five nearest neighbours was reached. Moreover, five nearest points had previously been used to define the cell size of a neighbourhood [65]. The spatial relationship was conceptualized as a fixed distance which was most commonly used to detect clusters for point features. It was in the interest of this study to focus on the influence within the neighbourhood. With this approach, points within the defined neighbourhood were assigned a value of 1 and a value of zero for points outside the neighbourhood (no influence). Row standardization was applied during the generation of the spatial weight matrix, as recommended for a potentially biased distribution. The spatial weight matrix was determined separately for Peninsular and East Malaysia for total, male, and female respondents. Sensitivity analysis conducted for fixed distances of 3 km and 5 km at 5, 10, and 30 nearest neighbors found no significant difference in the value of the Moran’s index. Figure 3 depicts the location of respondents in rural and urban settings, underscoring the necessity of an 8 km buffer distance or five nearest neighbours.

### Multilevel linear regression for BMI estimation

As cluster randomization was conducted at the enumeration block level in the NHMS 2015 and MANS 2014, multi-level linear regression was used to produce BMI estimates. This method was used instead of sampling weights to control for survey bias as we considered each respondent’s location as an individual point location. Multilevel linear regression was applied to account for survey bias and unexplained variability in the clustering of respondents at the enumeration block level. The final number of enumeration blocks is 1143. The analysis provided a more accurate estimate by correcting for underestimated standard errors, which could lead to an overstatement of statistical significance. The intraclass correlation coefficient (ICC), calculated as the ratio of the between-cluster variance to the total variance, indicated that the percentage of BMI variation attributed to clustering at the enumeration block was 4.0%. Maximum likelihood with robust estimation was applied to handle violations of the model assumptions. Robust standard errors were recommended in cases of unequal clustering groups and in the presence of a ICC, which may lead to biased estimates [66].

Each model was adjusted for the variables to produce estimates which were independent of the variable. Model 1 was adjusted for age to produce BMI independent of age. Model 2 was additionally adjusted for ethnicity. Model 3 was additionally adjusted for individual socioeconomic status such as marital status, education level, occupation, and income level. Finally, Model 4 was additionally adjusted for fast food proximity, location urbanity, district median household income and district population density to estimate their influence on the spatial clustering of BMI.

The formula for each model was represented as follows:

Formula for null model:

$$Y_{ij} \, = \,\beta_{0} \, + \,\gamma_{1} Z_{1ij} \, + \,u_{0j} \, + \varepsilon_{ij}$$

Formula for model 1:

$$BMI_{ij} \, = \,\beta_{0} \, + \,\left( {\beta_{1} \, \times \,Age_{ij} } \right)\, + \,\left( {\gamma_{1} \, \times \,Z_{1ij} } \right)\, + \,u_{0j} \, + \varepsilon_{ij}$$

Formula for model 2

$$BMI_{ij} \, = \,\beta_{0} \, + \,\beta_{1} \, \times \,Age_{ij} \, + \,\beta_{2} \, \times \,Ethnicity_{ij} \, + \,\gamma_{1} \, \times \,Z_{1ij} \, + \,u_{0j} \, + \varepsilon_{ij}$$

Formula for model 3:

$$\begin{aligned} BMI_{ij} \, & = \,\beta_{0} \, + \,\beta_{1} \, \times \,Age_{ij} \, + \,\beta_{2} \, \times \,Ethnicity_{ij} \, + \,\beta_{3} \, \times \,Marital{\kern 1pt} Status_{ij} \, + \,\beta_{4} \, \times \,Education{\kern 1pt} Level_{ij} \\ & + \,\beta_{5} \times Occupation_{ij} + \beta_{6} \times Income{\kern 1pt} Level_{ij} + \gamma_{1} \times Z_{1ij} + u_{0j} + \varepsilon_{ij} \\ \end{aligned}$$

Formula for model 4:

$$\begin{aligned} BMI_{ij} & = \beta_{0} + \beta_{1} \times Age_{ij} + \beta_{2} \times Ethnicity_{ij} + \beta_{3} \times Marital{\kern 1pt} Status_{ij} + \beta_{4} \times Education{\kern 1pt} Level_{ij} \hfill \\ & + \beta_{5} \times Occupation_{ij} + \beta_{6} \times Income{\kern 1pt} Level_{ij} + \beta_{7} \times Fast{\kern 1pt} Food{\kern 1pt} Proximity_{ij} \hfill \\ & + \beta_{8} \times Location{\kern 1pt} Urbanity_{ij} + \beta_{9} \times District{\kern 1pt} Median{\kern 1pt} {\kern 1pt} Household{\kern 1pt} Income_{ij} \hfill \\& + \beta_{10} \times District{\kern 1pt} Population{\kern 1pt} {\kern 1pt} Density_{ij} + \gamma_{1} \times Z1_{ij} + u_{0j} + \varepsilon_{ij} \hfill \\ \end{aligned}$$

where; *BMI*_*ij*_ is the body mass index for individual *i* in district *j*. *Age*_*ij*_ is the age of individual *i* in district *j*. *Et*ℎ*nicity*_*ij*_ is the ethnicity of individual *i* in district *j*. *MaritalStatus*_*ij*_ is the marital status of individual *i* in district *j*. *EducationLevel*_*ij*_ is the education level of individual *i* in district *j*. *Occupation*_*ij*_ is the occupation of individual *i* in district *j*. *IncomeLevel*_*ij*_ is the income level of individual *i* in district *j*. *FastFoodProximity*_*ij*_ is the proximity of fast food to individual *i* in district *j*. *LocationUrbanity*_*ij*_ is the urbanity of the location of individual *i* in district *j*. *DistrictMedianHouse*ℎ*oldIncome*_*ij*_ is the median household income of district *j*. *DistrictPopulationDensity*_*ij*_ is the population density of district *j*. *Z*_1*ij*_ is the value of the fixed effect for district *j* that individual *i* belongs to. β_0_ is the intercept (the value of *BMI* when all predictor variables and the effect of the enumeration block are zero). β_1_,β_2_,…,β_10_ are coefficients representing the effects of age, ethnicity, marital status, education level, occupation, income level, fast food proximity, location urbanity, district median household income, and district population density on *BMI*, respectively. γ_1_ is the coefficient representing the effect of the enumeration block (district) on *BMI*. *u*_0*j*_ represents the random effect at level *j* (e.g., district-level intercept). ϵ_*ij*_ represents the error term, which captures the difference between the observed BMI and the BMI predicted by the model for individual *i* in district *j*.

### Global Moran’s index

The Global Moran’s *I* statistic was applied to test the general tendency for high values to be located adjacent to high values across the entire spatial domain to generate one summarized measure. Relatively high estimates of Moran’s I indicated a spatially clustered dataset, whereas relatively low negative values indicated that the dataset was spatially dispersed. Statistical significance was set at z-score ≥ 1.96 and p-value < 0.05. Global Moran’s I statistic was conducted for the predicted BMI produced from the null model, Model 1, Model 2, Model 3 and Model 4 which was produced through multi-level linear regression. A decreasing Moran’s index of the adjusted BMI indicated that the spatial clustering was attributed to the adjusted variable. Sex-stratified analysis was conducted to observe the differences in the spatial clustering of BMI between males and females. BMI was regressed separately by male and female. The generated spatial weight matrix (fixed distance relationship at 8 km or five nearest neighbours) was applied in the test for each respondent group.

### Local indicator of spatial autocorrelation (LISA)

The local indicator of spatial autocorrelation (LISA) or Local Moran’s statistic evaluated the existence of local clusters and measured whether the value was closer to the values of its neighbours. It decomposed the global measurement (from Global Moran’s I) into contributions for each geographic region; therefore, the sum of the LISAs for all observations was proportional to the global indicator of spatial association. It also identified where the clusters were located and what type of spatial autocorrelation occured when outliers were identified [67]. The local spatial autocorrelation analysis resulted in five categories, where (i) ‘High-High’ indicated clustering of high values (positive spatial autocorrelation) (ii) ‘Low–High’ indicated that low values were surrounded by high values (negative spatial autocorrelation) (iii) ‘Low-Low’ indicated clustering of low values (positive spatial autocorrelation) (iv) ‘High-Low’ indicated an outlier where high values were surrounded by low values (negative spatial autocorrelation) and (v) ‘Not Significant’ indicated that there was no spatial autocorrelation. The analysis used BMI of Model 3 to determine the spatial clustering of BMI, which was not attributed to individual variation. The resulting spatial cluster map illustrated the local spatial patterns of significant positive (high-high) and significant negative (low-low) local spatial autocorrelation, whereas other points were hidden to avoid overcrowding the map. The generated spatial weight matrix (fixed distance relationship at 8 km or five nearest neighbours) was similarly applied. The false discovery rate (FDR) and 999 permutations with pseudo p-values < 0.001 were appropriately applied.

## Result

### Respondent’s characteristics

Total respondents was 17,158 persons (51.9% female, 48.1% male) with mean age of 38 years and 60.4% were of Malay ethnicity. Most of the respondents were married (69.3%), completed secondary-level education (76.4%) and half of them were employed by the government or private sector (49.7%) Geographically, majority were from Peninsular Malaysia (81.3%), middle-income districts (70.6%) and urban locality (58.6%), though 38.2% were from most populated districts. The median distance of fast-food proximity was 2.6 km, where 25% lived within the distance of 1 km or less and another 25% resided at 7.8 km or beyond from the nearest fast-food restaurant. Females were older than males. A larger percentage of females were unemployed (33.0% vs 3.7%) and in the lowest income bracket (21.4% vs 17.6%) compared to males who largely reported to be a private sector employee and self-employed. However, there was no significant difference in the distribution of male and female across urbanity, fast-food proximity or district median income categories (Table 1).

**Table 1:** Characteristics of all respondents by sex

Characteristics	All (N = 17,158)	Males (n = 8255)	Females (n = 8903)	*p-value*^*b*^
Age (years); Mean ± SD	38.0 ± 11.9	37.4 ± 12.0	38.6 ± 11.8	< 0.001
Ethnicity^a^				
Malay	10,368 (60.4)	4951 (60.0)	5417 (60.8)	< 0.001
Chinese	2391 (13.9)	1175 (14.2)	1216 (13.7)	
Indian	1179 (6.9)	542 (6.6)	637 (7.2)	
Indigenous groups	1957 (11.4)	890 (10.8)	1067 (12.0)	
Others	1263 (7.4)	697 (8.4)	566 (6.4)	
Marital status^a^				
Not married	5273 (30.7)	2709 (32.8)	2564 (28.8)	< 0.001
Married	11,885 (69.3)	5546 (67.2)	6339 (71.2)	
Occupation^a^				
Government	2312 (13.5)	1162 (14.1)	1150 (12.9)	< 0.001
Private	6206 (36.2)	3787 (45.9)	2419 (27.2)	
Self-employed	3717 (21.7)	2322 (28.1)	1395 (15.7)	
Unemployed	3250 (18.9)	309 (3.7)	2941 (33.0)	
Unknown	1673 (9.8)	675 (8.2)	998 (11.2)	
Education level^a^				
Tertiary	4330 (25.2)	2013 (24.4)	2317 (26.0)	< 0.001
Secondary	8778 (51.2)	4390 (53.2)	4388 (49.3)	
Others	4050 (23.6)	1862 (22.4)	2198 (24.7)	
Income^a^				
Q1	3366 (19.6)	1457 (17.6)	1909 (21.4)	< 0.001
Q2	3326 (19.4)	1634 (19.8)	1692 (19.0)	
Q3	3428 (20.0)	1761 (21.3)	1667 (18.7)	
Q4	3150 (18.4)	1561 (18.9)	1589 (17.8)	
Q5	3265 (19.0)	1670 (20.2)	1595 (17.9)	
Zone^a^				
Peninsular	13,948 (81.3)	6764 (81.9)	7184 (80.7)	0.036
East Malaysia	3210 (18.7)	1491 (18.1)	1719 (19.3)	
Urbanity^a^				
Rural	7110 (41.4)	3417 (41.4)	3693 (41.5)	0.908
Urban	10,048 (58.6)	4838 (58.6)	5210 (58.5)	
District population density^a^				
Least	5957 (34.7)	2903 (35.2)	3054 (34.3)	0.001
Medium	4645 (27.1)	2124 (25.7)	2521 (28.3)	
Most	6556 (38.2)	3228 (39.1)	3328 (37.4)	
District median HH income^a^				
Low	1822 (10.6)	830 (10.1)	992 (11.1)	0.113
Lower middle	6616 (38.6)	3191 (38.7)	3425 (38.5)	
Upper middle	5496 (32.0)	2653 (32.1)	2843 (31.9)	
High	3224 (18.8)	1581 (19.2)	1643 (18.5)	
Fastfood proximity				
Median (Q1, Q3)	2.6 (1.0, 7.8)	2.6 (1.0, 7.9)	2.6 (1.0, 7.7)	0.649

^a^All values were expressed as n (%) except where otherwise indicated

^b ^p-values indicated significant difference between males and females

### General clustering of BMI

The body mass index (BMI) of respondents was a mean of 26.0 and 25.5 kg/m^2^ from Peninsular and East Malaysia, respectively, where females had higher BMI than males at both regions (Peninsular: 26.5 vs 25.5 km/m^2^; East Malaysia: 25.8 vs 25.1 km/m^2^). Global Moran’s index of BMI among all respondents before adjusting for individual characteristics was higher in East Malaysia (Moran’s index = 0.39) than in Peninsular (Moran’s index = 0.55), which was greatly attenuated after adjusting for sex and age in Model 1. Further adjustment for ethnicity (Model 2), marital status, income level, education level and occupation (Model 3) and environmental variation (Model 4) sees a gradual reduction of spatial clustering, resulting in Moran’s index of 0.13 at Peninsular and 0.15 at East Malaysia. The spatial clustering of males was greater than that of females at Peninsular (Moran’s index = 0.21 vs 0.12) and East Malaysia (i = 0.25 vs 0.09) after adjusting for all factors (Model 4). Notably, the clustering of females decreased significantly in Model 1, highlighting age as a prominent factor for spatial clustering. Conversely for males, Moran’s index also decreased in Model 3, signifying the role of marital status, income level, education level and occupation in the spatial clustering of their BMI. Furthermore, the Moran’s index of BMI in Model 4 remained significant, indicating that fast-food proximity and other environmental factors did not fully explain the general clustering of BMI (Table 2).

**Table 2:** General clustering of BMI in Peninsular and East Malaysia

	All	Male	Female
Peninsular Malaysia	(n = 13,948)	(n = 6764)	(n = 7184)
	Moran’s index (z-score) ***		
BMI^a^			
Null model	0.39 (248.2)	0.42 (131.2)	0.33 (108.8)
Sex-adjusted	0.29 (184.9)	NR	NR
Model 1^b^	0.15 (94.23)	0.26 (79.51)	0.09 (28.99)
Model 2^c^	0.14 (90.06)	0.23 (72.22)	0.12 (39.28)
Model 3^d^	0.12 (77.21)	0.19 (59.50)	0.11 (37.15)
Model 4^e^	0.13 (79.53)	0.21 (66.35)	0.12 (39.92)

East Malaysia	(n = 3210)	(n = 1491)	(n = 1719)
	Moran’s index (z-score) ***		
BMI^a^			
Null model	0.55 (116.2)	0.62 (66.06)	0.42 (50.52)
Sex-adjusted	0.38 (80.04)	NR	NR
Model 1^ b^	0.17 (37.63)	0.31 (33.15)	0.09 (10.48)
Model 2^c^	0.17 (36.55)	0.32 (33.98)	0.12 (13.87)
Model 3^d^	0.15 (32.01)	0.22 (23.77)	0.11 (13.08)
Model 4^e^	0.15 (31.78)	0.25 (26.89)	0.09 (11.22)

^a^ BMI was controlled for data clustering at enumeration block

^b^ Model 1 additionally adjusted for age only

^c ^ Model 2 additionally adjusted for ethnicity

^d^ Model 3 additionally adjusted for socioeconomic status

^e^ Model 4 additionally adjusted for fast-food proximity, location urbanity, district population density, district median household income

NR = Not relevant; *** All Moran’s index values were significant at p < 0.001

### Spatial clusters of high-BMI and low-BMI

**Fig. 4:**
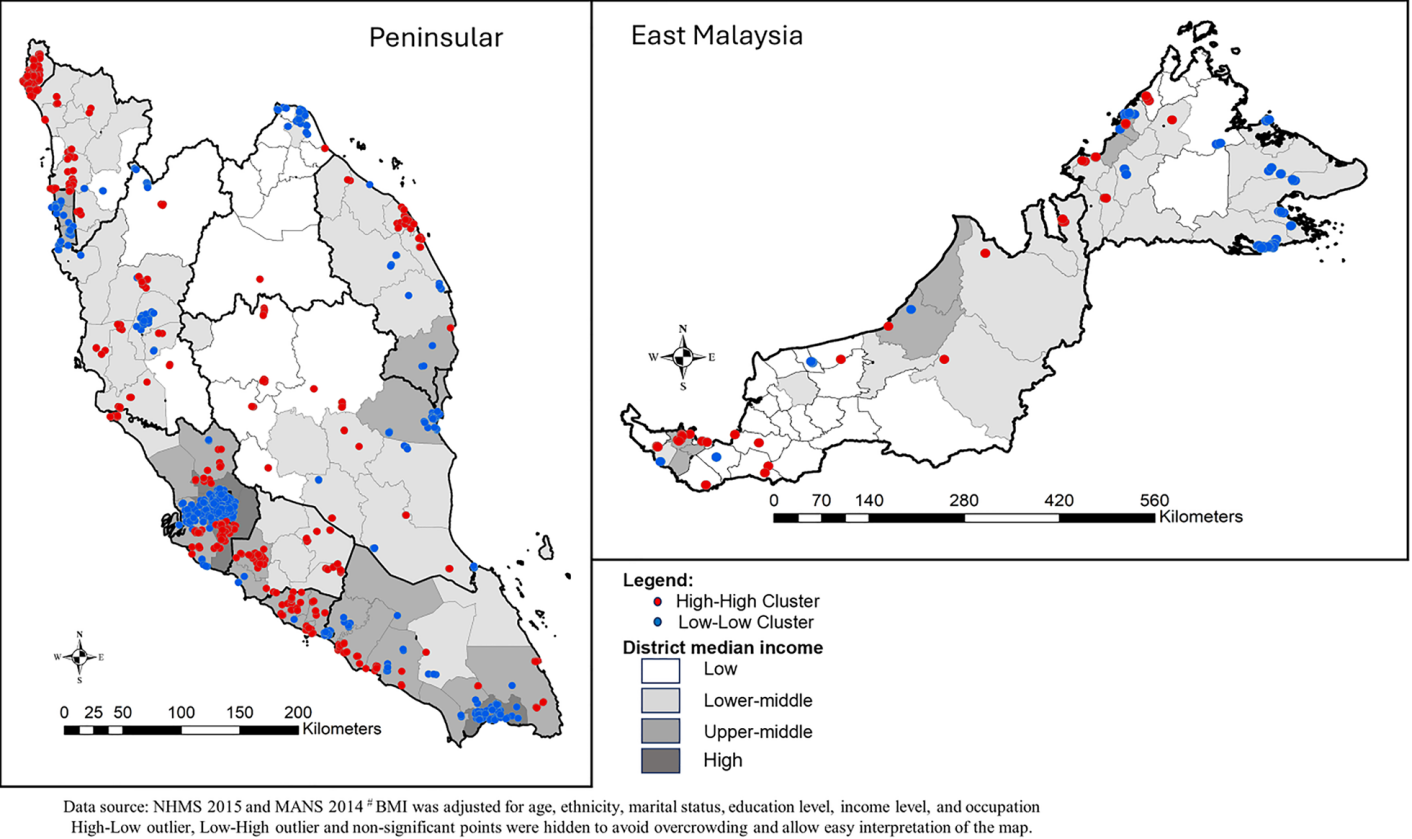
Spatial clustering of body mass index for all respondents at Peninsular and East Malaysia

The location of High-High clusters and Low-Low clusters of BMI after adjusting for individual factors (Model 3) signified spatial clusters that were independent of individual sociodemographic. At Peninsular, Low-Low clusters were located at higher income districts, whilst High-High clusters were located across most suburban districts. At East Malaysia, High-High cluster was dispersed across the southern and northwest region (Fig. 4).

**Fig. 5:**
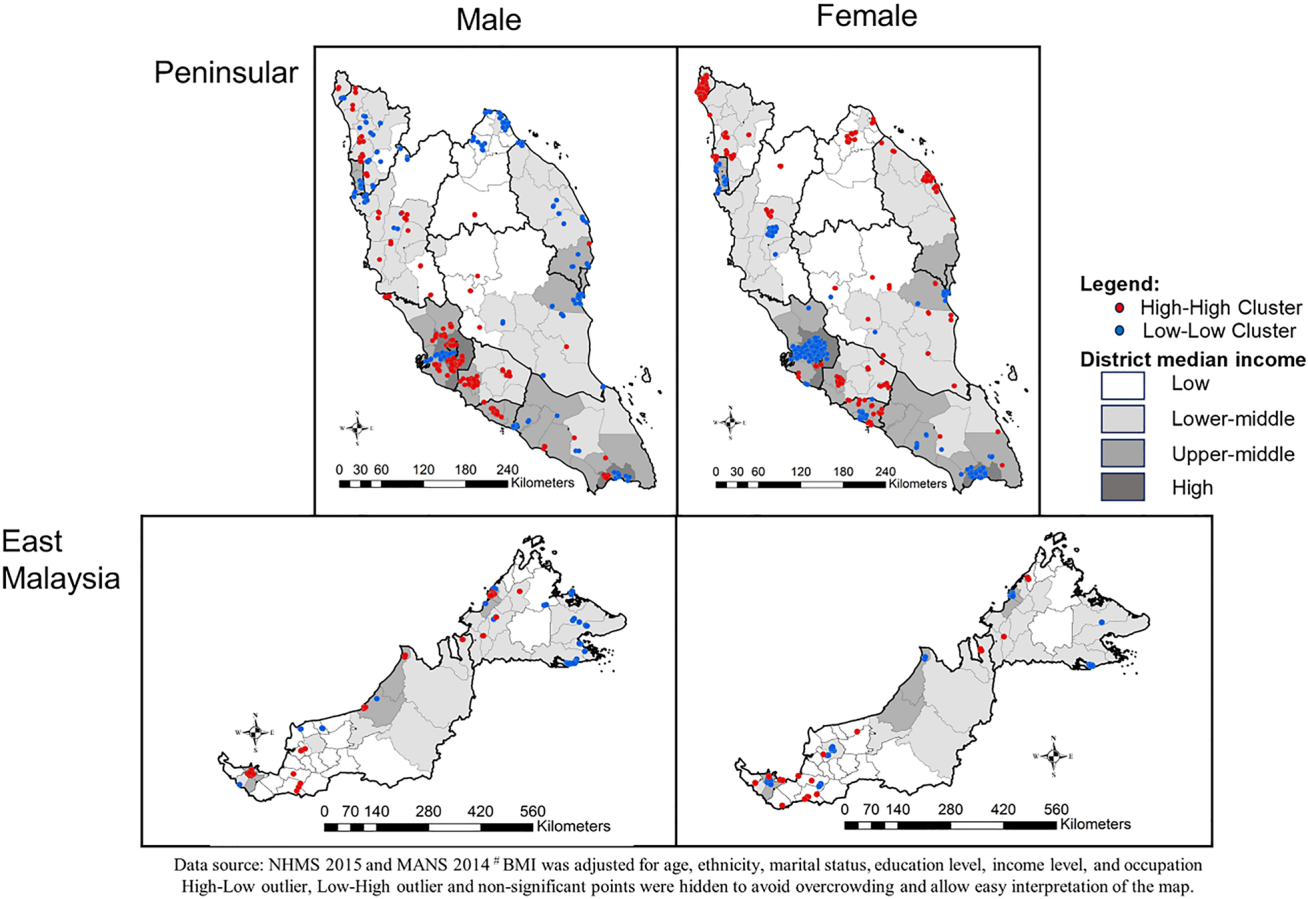
Spatial clusters of body mass index for male and female at Peninsular and East Malaysia

Overall, there were differences in the location of spatial clusters of BMI between males and females. High-High cluster for males was found in more developed areas, whilst High-High clusters for females were found in less developed towns, especially at northern Peninsular. In contrast, Low-Low cluster for females were found at more developed areas, whilst Low-Low cluster for males were at less developed areas. High-High clusters for males were also found mostly on the west coast of Peninsular, which has a more developed economy. However, there were some areas that demonstrated similar High-High and Low-Low cluster for both males and females. For example, the East Coast of northern East Malaysia were Low-Low cluster, whilst the southern West coast of Peninsular were High-high cluster for both males and females (Fig. 5).

## Discussion

This study demonstrated the presence of obesogenic environment through the significant spatial clustering of body mass index, independent of individual sociodemographic. The application of spatial weight matrix enabled the identification of clusters through the use of point locations of each respondent from nationwide population survey. Even though the exact prevalence estimates of a district were not derived, but the location of hotspots indicated that the area and its neighbouring locations had a higher BMI relative to the general mean. In general, high-BMI clusters were mostly located in the western Peninsular, which is more urbanized than the East Coast Peninsular. Specifically, only certain suburban areas were identified as high BMI clusters, which requires further understanding of the environmental factors involved. Studies from western countries found that obesity clusters were located in more rural areas, non-metro counties, and places with lower population density, intersection density, and diversity of facilities [40, 44, 68]. In comparison, higher prevalence of obesity was found in urban settings of China and India, reflecting global trends of increasing urbanization and its impact on lifestyle and dietary habits [50, 51]. National surveys in Malaysia have reported no differences in the prevalence of obesity between urban and rural localities or across socio-economic statuses [29]. However, the definitions of urban and rural areas that are usually applied in National Health Surveys cannot distinguish and detect suburban areas. Therefore, findings from the present study signified the importance of a spatial clustering analysis in identifying the geographical pattern of obesity, instead of relying on a mere urban–rural divide.

The present study highlighted a potential presence of obesogenic food environment in certain areas. The distribution of fast-food outlets was spatially dispersed, with KFC notably concentrated in downtown area and McDonald’s situated in the vicinity of a university campus, whilst certain less populated areas have fewer supermarkets and rely more on smaller grocery stores, convenience stores, and traditional markets [69, 70]. The existence of suburban sprawl was driven by government policies prioritizing foreign investment, large-scale projects, and domestic automobile manufacturing. Fragmented governance, middle-class affluence, and the dominance of a few local developers contributed to the sprawl, along with disjointed municipal zoning and transport planning [71]. However, these suburban districts suffer from inadequate provision of recreational facilities and playgrounds, lacking modern amenities [72]. Conversely, the urban parks within Kuala Lumpur city were generally perceived as safe, with a majority of visitors willing to contribute to their management [73, 74]. Physical activity at the suburban districts were confronted with unsafe environments characterized by congested roads, presence of stray animals, and crime [75]. Consequently, the use of public parks were minimal because the allure of public parks is predominantly influenced by their aesthetic appeal and perceived safety rather than their size [76]. The rapid economic development has facilitated widespread private vehicle ownership, leading to traffic congestion, air pollution, noise pollution, and a rise in road accidents [77, 78]. This surge in motorized transportation has resulted in underutilized and degraded pedestrian spaces, thereby diminishing the overall walkability of these areas [79]. In the suburban district of Sibu, walkability rated at an average score of 65%, despite high connectivity and relatively wide and clean pedestrian walkways. However, issues regarding pavement quality, shading, and resting areas detract from the comfort and safety of walking routes, thus discouraging pedestrian usage [80]. Even in Kuala Lumpur City Center, walkability levels, a composite of factors such as connectivity, land use diversity, comfort, security, and pedestrian access to rail transit stations, were average [81]. The lack of safety and connectivity were pivotal factors influencing walkability and the utilization of public transportation in this country [82].

We found that the spatial clustering of BMI among male adults in Malaysia was greater than that among females. A greater tendency to cluster indicated that the weight status of males was more likely to be affected by the local environment. In contrast, previous studies in South Korea and the US have reported greater spatial clustering among females, driven by occupation, physical activity, transportation, or nutrition policies with different impacts between the sexes [48, 49]. We also highlighted that the obesity clusters of males and females differed at some locations. Pertinently, urban districts were high-high clusters for males but low-low clusters for females. Historically in Southeast Asia, females were tasked with both familial caregiving and economic responsibilities in agriculture and trade, leaving little time for exercise or socializing [83]. Individuals with limited social engagement and lower income were more prone to obesity [84]. Unsafe environments also hindered females’ participation in outdoor activities and active transportation [76]. In contrast, males often face societal pressure to prioritize their professional and financial obligations, potentially fostering sedentary behaviors and unhealthy dietary choices. Individuals with irregular work hours, shift work, or high levels of stress are susceptible to disruptions in sleep patterns and unhealthy diet behaviour, which can contribute to elevated rates of obesity [85]. Moreover, males typically consume larger meal portions and exhibit a preference for calorie-dense foods, such as fast-food options [86; 87; 88; 89]. Males who predominantly work away from home commonly eat out with colleagues, increasing exposure to the food environmental influences [90]. This trend was also apparent in China, where working-age males from middle-income households who were initially lean had a rapidly expanding waistline [91]. Therefore, it is imperative to consider gender roles and gender-specific barriers in developing obesity prevention strategies. Interventions focusing on women may prioritize home-based exercises or cooking demonstrations and initiatives to increase access to affordable and nutritious foods, while those targeting men may emphasize workplace wellness programs or outdoor recreational activities.

Socioeconomic variation such as education level, income level, marital status, and occupation type had greater effect on the spatial clustering of males. This finding was in concordance with the evidence that obesity in males were more affected by socio-economic status, compared to females. [18]. Residents of a lower-income neighbourhood in Kuala Lumpur city with relatively higher income, were more likely to have lower BMI [17]. In a mixed-income neighbourhood, socioeconomic inequalities can influence the lifestyle behaviour leading to obesity. Lower socioeconomic individuals commonly rely on readily available, cheaper yet less nutritious food options. Due to financial instability and environmental stressors, individuals in lower-income brackets experience increased chronic stress, which can result in unhealthy coping mechanisms such as overeating and sedentary behaviors, further contributing to obesity. Consequently, it may be inferred that individuals with low incomes who reside in environments conducive to obesity would be most affected. In a review of the associations between built environments and obesity was usually stronger among those with lower socioeconomic status [92]. However, the complex relationship between environment and obesity varies across populations. In Netherlands, the association of obesogenic environment was stronger in younger adults, females, high income and those who live in highly urbanized areas and with high neighbourhood socioeconomics [93]. Similarly, adults living in the highest tertile of obesogenicity was associated with higher odds of developing cardiovascular disease [94]. Access to park connectors in Singapore was found to only decrease the BMI of higher socioeconomic status females [95]. A local study revealed that living in a neighbourhood with access to active transportation and high socioeconomic status, was associated with a lower prevalence of obesity [84]. Consequently, the extent to which an obesogenic environment exerts its impact depends on the type of environment and the individuals who are most susceptible to and exposed to it.

Considering the data collection period of 2014 and 2015, it is possible that changes have occurred in the spatial clustering of BMI. In recent, urban development plans have included addition and conservation of green spaces, enhanced security in high-density areas, improved public transportation, and providence of e-government services [96]. However, these initiatives aiming to create environments that support healthy living by promoting walkability, active transportation, and access to recreational facilities were only at selected districts. Majority of population are living in suburban sprawls, with an increase in fast-food restaurants and food delivery services. Various health promotion policies and campaigns may have increased the awareness of healthy eating, but the availability of healthier food options and the preference for it are still limited. Furthermore, there has been an expansion of fast-food chains, dessert stores, and convenience stores offering sweetened, calorie-dense, processed foods. Food delivery services have also made it convenient for people to obtain food out-of-home without traveling and encouraging over-consumption of non-nutritious food, as nutrients are lost during the delivery process. This shift in the food environment could contribute to an obesogenic environment by promoting unhealthy eating behaviours. Therefore, there is a need for continuous monitoring of the environment using the spatial methodologies discussed in this study, possibly incorporating the food environment matrix as part of the smart city initiatives.

The spatial clustering persisted despite controlling for environmental factors such as fast-food proximity, district median household income, district population density and location urbanity, implying a limited contribution of these factors to the spatial clustering of obesity. Past literatures had documented a generally very small effect of environment on weight status, with high risk of bias [92, 97]. In a large cross-sectional population survey in the Netherlands, the obesogenic index explained only 0.05% variance in BMI with a largely inverse and non-linear association, mostly driven by the food environment measures [93]. The association between fast-food environment studies and health outcome were inconsistent, where positive, null and negative associations were found among different studies conducted in Netherlands [98–101]. Moreover, another study revealed that dietary behaviours only mediated about 3% of the total effect of obesogenic environment and health outcomes [94]. The obesogenic environments are rather upstream determinants of health, with complex pathways between the built environment exposure and downstream BMI, involving multiple known and unknown mediators and modifiers.

We acknowledge that having more information on the environment will enhance the knowledge of the obesogenic environment. However, other studies have demonstrated that a larger obesogenic environment index with more components and more parameters might not always explain more variance than a smaller index with fewer components. Food environment components had approximately the same explained variance with the overall obesogenic index [93]. In fact, the sub-indices of the index perform better than the overall index in terms of explaining health outcomes [102; 103]. Dalmat and colleagues also revealed that simple walkability proxies such as population density could reasonably predict walking compared to more complex composite indicators [104]. Housing price as an indicator of area socioeconomic status improved the direction of association and model fit of obesogenic index, performing better than a composite index with 17 components [93]. Concordantly, area socio-economy status explained most of the geographical variation in obesity prevalence in Western countries after accounting for population density, race/ethnicity and age [54; 105]. Hence, this study postulated that the current parameters would be a sufficient proxy for a nationwide study. Further obesogenic studies could also explore on parameters such as convenience stores, supermarkets, sport facilities, public parks, including food stalls, street vendors, and small groceries of which a large portion of Malaysians obtain their food from, as it might require strenuous ground-truthing activities which was more suitable as a case study.

The strength of this study lies in the novelty of the spatial weights applied to single-point analysis. The method used in this study allowed the analysis of sparse and irregular locations of survey respondents across geographical borders. In addition, the area of influence or neighbourhood for each respondent was considered to vary instead of assuming that all respondents in the same district were exposed to the same area of influence. This method could avoid the modifiable areal unit problem (MAUP) which may occur when using administrative boundaries [106]. The spatial weight applied in this study could be integrated into public health planning and evaluation, especially in routine surveillance activities. This method makes a huge contribution to the field of health monitoring especially when data are insufficient to estimate values at smaller geographical area boundaries for public health attention at the local level. Spatial clustering analysis have been employed to assess geographical patterns of Urban Vitality Index [107], Urban Housing Wellness Index [108], urban environmental quality [109], urban quality of life [110] and Urban Social Vulnerability Index [111]. These highlighted the potential of the analysis in informing public health interventions and further exploration on the local environments to improve health of population in developing countries.

Several limitations are acknowledged in this study. Firstly, as a secondary analysis, this study was confined to the limits of the NHMS 2015 and MANS 2014. The sampling of respondents for the national surveys was randomized at enumeration blocks. Therefore, findings from this study could only refer to the enumeration blocks sampled and should be generalized to other areas with caution. National household surveys also was commonly biased towards the lower income population and those who were unemployed [112]. These affect the spatial autocorrelation bias to the lower income population. However, the strength of the survey outweighs the limitations. There was a well representation of respondents in terms of urbanity, region, and most of the districts as this survey was designed to represent the actual populations across the country. In addition, the weight and height of respondents was reliable as it was measured by trained research facilitators. Thus, study findings should be an indication on the existence of obesogenic environment which require further exploration. Beyond the Malaysian context, this findings highlight the possibility of identifying obesogenic environment within the limits of data availability and the importance of studying the effect of suburban sprawl.

Furthermore, there was a temporal inconsistency between the NHMS 2015 and MANS 2014 and the fast-food restaurants sampling in the year 2018. Due to the failure to obtain the address of fast-food restaurants registered in 2015 from local administrative council business registers, the only viable method at the time of study was to access the official websites of fast-food franchises. To minimize temporal discrepancies, manual inspection was performed to identify outlets that opened after 2015. This approach led to the third limitation of the study, which was the sampling of only selected large fast-food restaurant franchises in Malaysia. Previous studies that lacked a comprehensive national food retail database have also utilized this method [113–115]. However, narrow construct definitions could provide more precise measures of the retail food environment, capturing a more consistent type of food provision and producing more positive associations than broader definitions [116]. Furthermore, large franchises have a greater impact on the food environment due to their larger buffer against economic downturns. Other food stores and restaurants often operates nearby a fast-food restaurant. Therefore, although this study may have excluded other franchised and non-franchised outlets selling fast food items, targeting selected large fast-food chains can be a good proxy of locations with many restaurants.

## Conclusion

In conclusion, this study revealed the presence of obesogenic environments in Malaysia, particularly in suburban areas, where obesity clusters varied between males and females at certain locations. The identification of obesity clusters emphasized the significance of considering environmental factors in the development of obesity. It would be cost-effective to conduct sex and geographically targeted interventions aimed at creating healthier food environments, particularly in areas where healthcare resources are limited. Local governments or public health officials can use this spatial information to establish a task force comprising various stakeholders to investigate the multifaceted aspects of the obesogenic environment and implement specific policy measures and community-level interventions that are well-suited for their local community. For example, introducing a nutrition measure for restaurants, increasing the availability of healthy food options, and increasing infrastructure to encourage walkways and public transportation. These efforts especially in suburban areas are more likely to maintain obesity prevention efforts over time and achieve population-level effects. Policymakers and public health professionals should incorporate spatial analysis into health surveillance activities to direct resources towards specific locations. Specifically, spatial clustering analysis should be made compulsory in all technical reports of health outcomes. Further research is warranted to understand the relationship between specific environmental factors and obesity at local district level.

## Data Availability

The data that support the findings of this study are available from the National Institute of Health, Malaysia but restrictions apply to the availability of these data, which were used with permission for the current study, and are not publicly available. However, the data are available from the National Institute of Health, Malaysia, upon request.
